# A systematic review of the use of virtual reality or dental smartphone applications as interventions for management of paediatric dental anxiety

**DOI:** 10.1186/s12903-021-01602-3

**Published:** 2021-05-07

**Authors:** Andrea Cunningham, Orlagh McPolin, Richard Fallis, Catherine Coyle, Paul Best, Gerald McKenna

**Affiliations:** 1 Centre for Dentistry, Queen’s University Belfast, Belfast, UK; 2 Queen’s University Belfast Medical Library, Queen’s University Belfast, Belfast, UK; 3 School of Social Sciences, Education and Social Work, Queen’s University Belfast, Belfast, UK; 4 Centre for Public Health, Institute of Clinical Sciences Block B, Royal Victoria Hospital, Queen’s University Belfast, Belfast, UK

**Keywords:** Virtual reality, Paediatric dentistry, Behavioural management, Pain control, Anxiolysis, Prevention, Autism spectrum disorder

## Abstract

**Background:**

Virtual reality (VR) has been used successfully in medicine both as a distraction tool during procedures, and as an acclimatisation tool to prepare for a procedure or experience. It has not yet become widely used in dentistry, but could theoretically have a role in exposure-based acclimatisation for dental experiences.

**Methods:**

To examine the use of VR or bespoke dental smartphone applications pre- or perioperatively in dentistry, to decrease anxiety in a paediatric population attending for dental examination or treatment, compared with children/adolescents who receive no intervention, or more conventional behavioural management techniques.

Searches were made of eight electronic databases: the Cochrane Oral Health Group’s Trials Register, The Cochrane Central Register of Controlled Trials (CENTRAL), MEDLINE(PubMed), EMBASE, PsycINFO, CINAHL, Scopus and Web of Science. Further searches reference cross‐checks were performed to identify studies that were not discovered online.

**Results:**

Systematic reviews and randomised control trials have demonstrated the successful use of VR to both distract patients perioperatively during medical procedures, and also preoperatively to prepare them for these interventions. However, to date, VR has only been applied to dentistry in a very limited number of studies. Three studies using virtual reality in a dental setting demonstrated decreased pain and anxiety compared with no intervention. All three of these studies were carried out in the perioperative period. A fourth study used a bespoke dental app and imagery to prepare patients with Autism Spectrum Disorder (ASD) for dental treatment, finding statistically significant decreases in both the number of appointments and number of attempts required to carry out a procedure.

**Conclusion:**

VR is a promising tool which to date has been under-utilised in dentistry. High quality, clinical studies are required to assess the use of preoperative VR and smartphone applications to prepare patients for dental examination and procedures under local or general anaesthetic.

## Background

In recent years, public awareness and use of virtual reality (VR) has grown, largely due to the gaming industry. VR can be described as “a computer generated, three-dimensional world in which the user interacts with virtual objects” or characters [1]. Naturally, this has segued into healthcare due to its immersive and entertaining nature. Some authors have described the applications of virtual reality in healthcare as either emotion- or problem-focused solutions. Emotion-focused interventions divert the user’s attention away from the underlying stressor, e.g. distracting children undergoing vascular access to lower their emotional response. In comparison, problem-focused interventions assist the user to overcome the stressor itself through content that aids preparation, mental rehearsal or guided imagery e.g. familiarising children with the process of having vascular access [2]. This can be considered an exposure-based approach. A key benefit of VR is that it can ‘transport’ users to a virtual environment, giving users a sense of ‘presence’. It may provide an effective avenue for an exposure-based treatment.

Dental anxiety is common with an estimated prevalence between 6 and 20% in children aged 4–18 years old [3]. In Northern Ireland, the Child Dental Health Survey found that parents reported moderate to extreme dental anxiety in 14–24% of children (stratified by age), and 62–68% of 12–15 year olds self-reported moderate to extreme dental anxiety [4].

Approximately 600,000 children in the UK each year require a general anaesthetic for surgery, medical treatment or a diagnostic procedure [5]. Approximately 50–75% of these children will have significant anxiety about this, with adverse consequences in both the short and long term including bedwetting, nightmares and anxiety [6, 7]. Tooth decay is still the most common reason for children aged 5 to 9 to be admitted to hospital [8], and one study reported 60% of dental GAs in Northern Ireland were carried out due to anxiety/fear [7]. Both the number of dental GAs and the number of extractions under GA are also increasing [9]. In Northern Ireland alone, more than 5100 children were admitted to hospital for tooth extractions in 2017. In 2016/17 dentists extracted 22,699 teeth, of which 88% were baby teeth, taken out due to decay [10].

Distraction has been successfully used in dentistry for many years, and works based on the assumption that pain perception has a large psychological component, in that if less attention is directed at a noxious stimuli, less pain is perceived. It follows that optimal distraction could be achieved from a multisensory experience such as virtual reality. VR can employ kinaesthetic stimuli by sensing movement of the head and hands, however this is not appropriate during dental treatment. Therefore, an exposure-based approach should be considered.

Health-related apps and wearable technology are revolutionising health and healthcare for patients, playing roles in patient education, data monitoring, symptom management, management of chronic disease, behaviour modification and preparation/acclimatisation [11]. With regards to anxiety, apps providing patient information combined with psychological techniques can be utilised as a home-based acclimatisation system that the patient can use on an ad hoc basis in the run up to their procedure. This is theorised to be particularly relevant for younger populations who have ‘grown up’ with these technologies [12]. Apps related to many aspects of general health are already widely available, but the volume of available apps is vastly outgrowing the body of evidence to support their efficacy. A recent systematic analysis of commercially available apps aiming to psychologically prepare patients for medical procedures identified five apps, however none had any evidence to support their efficacy [11]. Only one of these apps targeted children with upcoming medical and dental procedures, aiming to reduce anxiety and improve recovery via a mixture of hypnosis, breathing exercises and guided imagery. This app is not currently available in the UK app store. None of these apps appeared to utilise VR.

The preoperative use of virtual reality and other technology to decrease anxiety is well-documented in the medical literature, including prior to general anaesthesia [13, 14]. A randomised control trial was carried out by Ryu et al. 2017, using an immersive virtual reality tour of the operating theatre for children before a general anaesthesia [15]. Increased preoperative anxiety is associated with poorer postoperative outcomes; including increased pain and analgesia dosage, longer recovery and further anxiety. It is also associated with increased maladaptive behaviours such as separation anxiety, bedwetting and sleep difficulties [16]. Children exposed to VR in the study had significantly lower preoperative anxiety than a control group, as well as increased compliance during induction [15].

Exposure therapy (ET) is considered by many as the first choice treatment for specific phobias [17]. While there is some debate regarding the underlying mechanisms, ET is a treatment designed to lessen the effect of feared stimuli through repeated and graded exposure. When one is unable to escape or avoid the feared stimuli and associated anxiety (fear) has decreased, a ‘process of habituation is said to have taken place’ [18]. Most recently, ET has been adapted for use within virtual environments (known as Virtual Reality Exposure Therapy – VRET) with studies showing that one can activate the same physiological and psychological responses as if “in the presence of the feared stimuli” [19, 20]. This may be due to the ability of VR to create a sense of presence and immersion within the user. Immersion is defined as “the degree [with] which the range of sensory channel is engaged by the virtual simulation” whereas presence is defined as “one’s sense of being in the virtual world” [21, 22]. High immersion within VR engages a variety of senses (visual, auditory, haptic) which can block out external sensory cues. The psychological sensation that results from this is known as presence. The interaction between these two concepts may explain the ability of VR to provoke a fear response, even though the user is aware that the environment is simulated. This is particularly relevant for groups that find it difficult to engage in psychological treatment or where traditional exposure based approaches are impractical. A systematic review by Botella and colleagues demonstrated Virtual Reality as a useful tool to improve ET although the expansion of this technology into routine clinical settings (while inevitable) is yet to occur [13].

A recent systematic review assessed the effect of technology-based preoperative preparation for medical procedures, and found anxiety was significantly reduced in children in 25 of 33 studies, and in parents in 11 of 33 [16]. Of these studies, one by Campbell et al. [23], looked at children preparing for dental treatment, although this was an interactive computer package or cartoon rather than virtual reality. The children were prepared immediately prior to their general anaesthetic, with images of the general anaesthetic experience and process. The study found that children had decreased preoperative anxiety versus a control group who were only verbally prepared, and it was noted the children in the experimental group had significantly more coping behaviours at induction and postoperatively. Of the 33 studies in this review, only one of them utilised virtual reality [24], compared with a dose of 0.3 mg/kg oral midazolam. No difference in heart rate or observer-rated anxiety was noted between the two, indicating preparation with virtual reality may have a comparable anxiolytic effect to midazolam, without the need for pharmacological intervention.

A literature review in 2005 by Wismeijer et al. looked at the use of virtual reality and audiovisual glasses as distraction measures/adjunct analgesic techniques in 20 studies in the perioperative period in medicine and dentistry, predominantly in adult patients. The authors found in all but one study anxiety decreased or remained unchanged [25]. They identified one study aimed at patients undergoing dental procedures, finding all pain measures decreased and that ‘presence’ was higher with VR, however the sample size was only n = 2 and both patients were over 50 years old [26]. No apps were utilised preoperatively. The results strongly suggested VR and AV distraction were very promising analgesic techniques, however the review criticised the methodology, sample size and lack of appropriate control conditions, concluding that further high quality studies were required.

It appears that to date, virtual reality is a largely untapped resource in dentistry, especially pre-operatively. The aim of this review is to identify studies applying virtual reality or bespoke smartphone applications to dentistry, either pre-operatively or peri-operatively, to decrease patient anxiety; and assess whether they have been effective.

## Methods

### Protocol and registration

This systematic review was conducted according to PRISMA guidelines [27]. The review protocol was registered with PROSPERO – the international prospective register of systematic reviews (reference CRD42019155570).

### Eligibility criteria

The focused PICO (participants, interventions, comparisons and outcomes) question for the systematic review was: Can the use of virtual reality or smartphone applications decrease dental anxiety in paediatric patients attending for dental examination or treatment, compared with no intervention or more conventional behavioural management techniques?

This also guided the study selection criteria (Table 1).

**Table 1:** PICO focus question, criteria for inclusion, sources of information, search terms, search strategy, search filters, and search dates

Focus question	Can the use of virtual reality or smartphone applications decrease dental anxiety in paediatric patients?	
Criteria	Inclusion criteria	English language Subjects < 18 years Involving dental treatment or examination Involving element of virtual reality or bespoke smartphone application Anxiety as primary/secondary outcome
	Exclusion criteria	Non-English language papers Adult patients (> 18) Non-dental studies Smartphone applications for uses other than to alleviate anxiety (e.g., to improve oral hygiene Studies using video glasses alone without virtual reality components
Information Sources	Electronic databases	The Cochrane Oral Health Group’s Trials Register, The Cochrane Central Register of Controlled Trials (CENTRAL), MEDLINE(PubMed), EMBASE, PsycINFO, CINAHL, Scopus and Web of Science
	Journals	All peer reviewed dental journals available online in above databases
	Other	Online internet search engine (i.e. Google), internet research community websites (https://www.researchgate.net/), reference crosschecks, hand-searches, etc
Search Terms (PICO)	Population	All humans studies involving Medical OR dental (medic* OR dentist* OR dental)
	Intervention/exposure	Treatment or first appointment (treatment* OR procedure* OR operation* OR "first appointment*" OR "first clinic*" OR "first attendance*" OR hospital* OR an*esthe*)AND preparation OR anxiety OR autism OR stress (prepar* OR anxi* OR autis* OR stress*)
	Comparison	None
	Outcome	Anxiety/stress
Filters	Language	English
	Species	Humans
	Ages	No filter
	Journal categories	All
Search query as performed in electronic databases	(TITLE-ABS-KEY (medic* OR dentist* OR dental)) AND ((TITLE-ABS-KEY ((prepar* OR anxi* OR autis* OR stress*)) AND TITLE-ABS-KEY ((treatment* OR procedure* OR operation* OR "first appointment*" OR "first clinic*" OR "first attendance*" OR hospital* OR an*esthe*)))) AND ((TITLE-ABS-KEY (({app} OR app's OR apps) AND (mobile* OR smartphone* OR iphone* OR ipad* OR tablet*)) OR TITLE-ABS-KEY ({little journey}) OR TITLE-ABS-KEY ({virtual reality MRI}) OR TITLE-ABS-KEY ({take ten}) OR TITLE-ABS-KEY ({sidekicks!}) OR TITLE-ABS-KEY ({brighthearts}))) AND (LIMIT-TO (LANGUAGE, "English")) AND (LIMIT-TO (DOCTYPE, "ar") OR LIMIT-TO (DOCTYPE, "re"))	
Search Dates		Last confirmatory online final search was performed on 31/08/2019. No further online searches were performed after this date

All English-language human studies reporting on children and adolescents (< 18 years) undergoing dental examination or treatment involving an element of virtual reality or a bespoke smartphone application were included in the review, whether preoperatively for preparation/acclimatisation, or perioperatively for distraction. Randomised and non-randomised trials were eligible for inclusion. Included studies must include anxiety as a primary or secondary outcome.

The exclusion criteria were studies including adults, non-dental studies, non-English language papers, smartphone applications for uses other than to alleviate anxiety (e.g., to improve oral hygiene) and studies using video glasses without associated smartphone applications aimed at anxiolysis, or without virtual reality components. No exclusions were made based on comparison groups.

### Information sources

An initial search of the PROSPERO database confirmed no ongoing or published systematic reviews looking at “virtual reality” and “dentistry”.

Eight electronic databases were searched: The Cochrane Oral Health Group’s Trials Register, The Cochrane Central Register of Controlled Trials (CENTRAL), MEDLINE(PubMed), EMBASE, PsycINFO, CINAHL, Scopus and Web of Science.

### Search strategy

The search strategy was designed by a clinician (AC) and a research librarian for Medicine, Dentistry and Healthcare Sciences (RF) after discussions with the remaining team members (GMcK, OMcP, CC, PB), see Table 1. It was purposely designed to retrieve a large number of results to ensure no relevant studies were missed.

The electronic search strategy was conducted on eight databases by combining subject heading terms with keywords and text words. Search terms were based on 5 main concepts:

1. Search terms related to medicine or dentistry (to ensure all reviews featuring dental treatment were captured)
2. Search terms related to preparation, anxiety, autism or stress
3. Search terms related to treatment, procedure, first appointment, hospital or anaesthetic
4. Search terms related to mobile/smartphone/tablet/iPhone/iPad apps
5. Limited to English results.

The search terms were then combined with an “OR,” and PICO categories were combined using “AND” to create a final logic search query. At this point, no effort was made to confine results to a paediatric population.

Further searches resulting from reference cross‐checks were performed to identify studies that were not discovered by searching the above databases.

The final search was carried out on 31th August 2019. The search strategy can be found in Appendix 1.

### Study selection

All relevant studies fulfilling the inclusion criteria were included in this review. After de-duplication, a list of 872 abstracts remained. A title and abstract screening was carried out independently by the first and last authors (AC, GMK) and a final list of studies was compiled of 23 papers for full text analysis. These studies were analysed by the first and last authors independently with any disagreements resolved via a consensus discussion with the rest of the group. Any uncertainty was discussed and resolved with a third author (OMcP). Other forms of technology identified such as videos, tablets, handheld devices, internet or web-based programmes, or video glasses without a virtual reality component were not included. The final list of studies to be included in the review was agreed between these investigators and data extraction carried out.

### Data extraction

The primary investigators (AC and GMK) extracted data from the included studies independently and were reciprocally blinded**.** Data extracted included author, year, study design, sample size, age, dental procedure, intervention used, timing of intervention, control/comparison groups, outcomes, and outcome measures.

During data extraction, for any uncertainty involving the extracted variable, a consensus was always reached by both investigators before finalising the extracted data. The data extracted from the included studies can be seen in the Table 2.

**Table 2:** Summary of results

Authors	Year	Study design	Period	Study Participants	Results in VR condition	Dependent variables	Conditions	VR Equipment	Dental Procedure
Al-Halabi [33]	2018	RCT	Periop	102 6–10 year olds	Tablet had best results in anxiety and pain	Wong-baker faces, pulse, behavious (FLACC)	Vs control vs tablet	VR box and Av glasses	IAN block
Aminabadi [34]	2012	RCT	Periop	120 4–6 year olds	SS decrease in pain and anxiety in VR group	Wong Baker FACES, MCDAS	Vs control	VR eyeglasses	Fluoride therapy then restorative treatment
Panda [35]	2017	RCT	Periop	30 6–8 year olds	SS less pain in VR group	Faces pain scale (revised)	Vs control	Virtual reality smartglasses, detachable earphones	Pulp therapy
Zink [36]	2018	RCT	Periop	40 9–15 year olds with ASD, first visit	SS decrease in number of attempts and number of appointments	Number of attempts to acquire skill, total number of attempts, number of appointments to have dental prophy	Vs control	App: Autistic Child Goes to the Dentist (no VR)	Dental prophylaxis

### Risk of bias

The potential level of bias in each study was assessed. As all included studies were randomised control trials, the Cochrane collaboration’s tool was used to assess the risk of bias [28]. These findings are presented in Table 3.

**Table 3:** Assessment of Risk of Bias of included studies

	Alhalabi [33]	Aminabadi [34]	Panda [35]	Zink [36]
Sequence generation	Low risk	Low risk	High risk	High risk
Allocation sequence concealment	High risk	High risk	High risk	High risk
Blinding of personnel	High risk	High risk	High risk	High risk
Blinding of outcome assessment	High risk	High risk	High risk	High risk
Incomplete outcome data	Unclear	Unclear	Unclear	Unclear
Selective reporting	Unclear	Unclear	Unclear	Unclear

## Summary measures

The primary outcome measure of this review was anxiety in paediatric patients, however in this population it can be difficult to differentiate from pain and anxiety/fear of pain. Anxiety was variably measured, via pulse rate or the Modified Child Dental Anxiety Scale (MCDAS) [29]. Pain was measured by Wong-Baker FACES [30], The Face, Legs, Activity, Cry, Consolability scale (FLACC) [31] and the Faces Pain Scale (Revised) [32].

Secondary outcomes assessed were the length of the appointment, number of appointments required to complete named treatment, and whether the child ultimately required sedation or a general anaesthetic to undergo treatment.

## Results

### Study selection

**Fig. 1:**
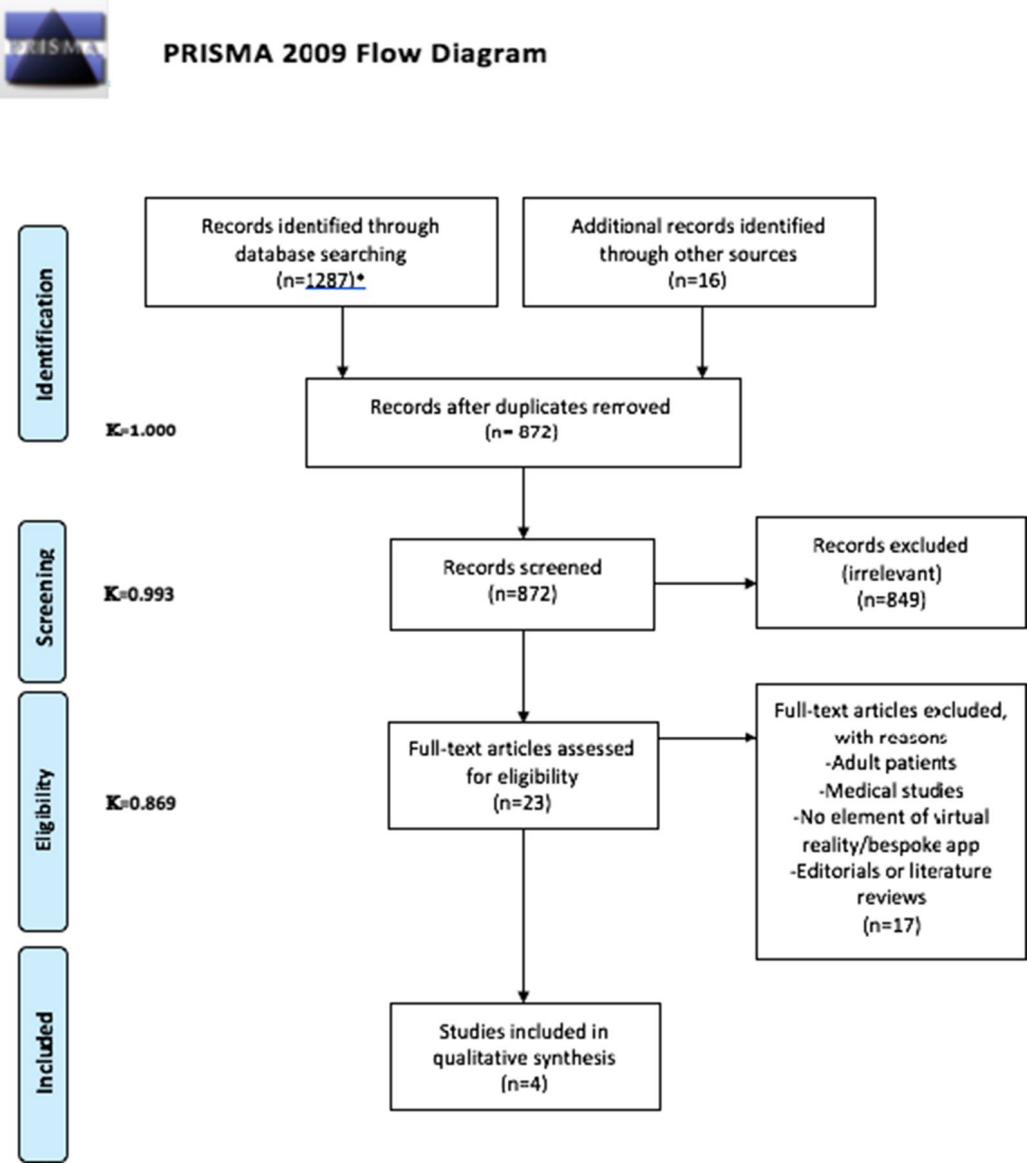
PRISMA search strategy

The search queries identified a total of 1287 abstracts from six different databases (see PRISMA flowchart). These results were de-duplicated, leaving a total of 856 unique abstracts after de-duplication. A further 16 abstracts were identified from reading the references of relevant articles. 872 abstracts were screened, and 849 records excluded as irrelevant to the review question. A total of 23 articles were selected for full-text analysis. From these, 19 articles were excluded as either pertaining only to adults, or not featuring virtual reality/smartphone app or dentistry. Reviews were not included but the studies included in any reviews were analysed as part of this stage. A final total of 4 studies were included in the review for data extraction. Although the primary focus of the review was virtual reality, a search was also made for any bespoke applications (also using imagery) for home-based acclimatisation. Studies pertaining to Picture Exchange Communication Systems (PECS) alone were not included, as although they did facilitate home-based preparation for dental treatment, their efficacy is well-established, and if there was no novel technological element associated they were not included in the review. The flow of the entire search and the article identification process is shown in Fig. 1.

It was not felt there were significant numbers of similar studies available to facilitate meta-analysis.

### Study characteristics

All included studies were randomised control trials [33–36]. Each dealt with the peri-operative period, with no study participants having access to virtual reality or the app beforehand/at home to prepare.

The sample sizes varied from 30–120. The target age ranges varied, with the youngest included being 4 years old, and the oldest 15 years old. The study by Zink looked at a population of slightly older children/adolescents (9–15 years) with autism spectrum disorder (ASD). Three of the studies features the use of virtual reality in dentistry as a distraction technique [33–35], and the remaining study featured a smartphone app, also using imagery, similar to having a PECS embedded in a smartphone application for on-demand use (but without VR) [36].

### Synthesis of results

Kappa (κ) statistics were calculated to confirm the inter-investigator agreement for the extracted data. (Fig. 1).

A total of 292 children/adolescents were included in these studies, with 92 exposed to virtual reality, 20 exposed to the bespoke smartphone app, 34 exposed to active distraction via a tablet, and 146 acting as control groups. The mean sample size was 73. Three studies compared one experimental group to one control group [34–36]. The study by Al-Halabi compared conventional behavioural management techniques to virtual reality eyeglasses to a tablet device [33].

As discussed in “Summary measures”, a number of measures were used between the studies, making direct comparison difficult. These measures can broadly be classified into pain measures and anxiety measures, however it is well known in a paediatric population these feelings can be interchangeable and act synergistically. The ‘behaviour’ parameter has been characterised as a measure of ‘pain’ in this review but it could equally be viewed as a measure of anxiety. Due to the limited data available, meta-analysis was not possible.

#### Virtual reality

##### Anxiety

Anxiety was measured in two studies featuring virtual reality via pulse rate, or the Modified Child Dental Anxiety Scale (MCDAS). It was not measured in the study by Panda.

Pulse rate was used by Al-Halabi, who found a statistically significant difference (*p* = 0.043) in pulse rate between the group watching a tablet versus patients treated with convention behavioural management techniques, but no significant difference between VR and the tablet or between VR and the control [33].

Aminabadi et al. used the MCDAS, with these authors finding a statistically significant (*p* < 0.001) decrease in anxiety scores with the use of VR distraction [34].

##### Pain

Overall, pain was measured in the three studies featuring virtual reality, by Wong-Baker FACES, The Face, Legs, Activity, Cry, Consolability scale (FLACC), and Faces Pain Scale (Revised).

In the studies by Al-Halabi and Aminabadi, Wong-Baker FACES was used [33, 34].

Al-Halabi found no statistical difference in Wong-Baker FACES between the VR, tablet and conventional behavioural management techniques groups, yet.

Aminabadi found statistically significantly less pain in the VR group vs. the control. This is summarised in Table 4.

**Table 4:** Studies assessing pain via Wong Baker FACES

Study	Wong baker VR	Wong baker control	*p* value
Al-Halabi [33]	Not reported	Not reported	P = 0.536
Aminabadi [34]	1.89 (± 0.65)	3.00 (± 0.81)	P < 0.0001

The only study to use the FLACC scale was that by Al-Halabi, which again found no statistical difference between VR, tablet and conventional behavioural management techniques [33].

Finally, using the Facial Pain Scale (Revised), Panda et al. reported significantly less pain observed in the experimental group experiencing VR distraction (*p* < 0.05) [36].

#### Smartphone app

The study by Zink was unique in both intervention and outcome measures,

the former being an app that facilitated patient-professional communication among individuals with ASD (‘Autistic Child Going to the Dentist’), and the latter being; number of attempts to acquire skill, the total number of attempts, and the number of appointments required to have dental prophylaxis. This was compared with the Picture Exchange Communication System (PECS) [36].

This study found statistically significant decreases in number of attempts to acquire the skill proposed and number of appointments (*p* < 0.05).

### Risk of bias within the studies

The quality of the four included randomised control trials was overall quite low, all demonstrating an overall high risk of bias in allocation sequence concealment, blinding of personnel, and blinding of outcome assessment. Risk of bias was assessed using the Cochrane Risk of Bias Tool [28] (Table 3).

## Discussion

The limited number of studies identified by this review indicate that as yet, VR has not become widely used in dentistry. It also appears from this review that there has not yet been a study assessing the use and efficacy of VR preoperatively to prepare and acclimatise patients prior to dental treatment or surgery. In the three studies using virtual reality perioperatively in a dental setting, decreased pain and anxiety were seen compared with no intervention. In the sole study investigating the use of a bespoke dental app (but not VR) to prepare patients for dental treatment, statistically significant decreases were seen in both the number of appointments and number of attempts required to carry out a procedure. However, the quality of the evidence is poor.

Limitations of the review include the paucity of available literature to study compared to medical procedures, and therefore the inability to carry out a meta-analysis.

The varied nature of indices used to measure pain and anxiety precluded direct comparison or meta-analysis.

Overall, studies assessing perioperative use of VR demonstrate decreased pain and anxiety versus no intervention, but it is possible this intervention is less effective than active distraction with a tablet. In the study by Al-Halabi, VR proved less effective than tablet distraction at decreasing pain and anxiety, but this may be explained by the fact that wearing a large ‘VR box’ over the face during dental treatment causing reduced visual field leading to a loss of control, which may indeed increase anxiety and apprehension [33]. Larger head-mounted displays may also cause discomfort when supine. It should also be noted that the kinaesthetic aspect of VR is not applicable to dental treatment as it is not appropriate for the patient to move their head perioperatively. It may therefore be suggested that VR may be more appropriate in the perioperative period, for example prior to a general anaesthetic or to acclimatise a child with ASD to their first dental visit.

Audiovisual distraction with video glasses and filmed modelling were not included in this review as their effectiveness is well-documented [37], but the purpose of this review was to assess whether the more immersive nature of VR could prove more effective. It is therefore important to carry out further studies comparing these interventions to preoperative and perioperative VR, and whether increased ‘presence’ leads to increased anxiolysis. ‘Presence’ is a major concept in the field of VR, indicating how immersed in the virtual environment the user is, or if they feel ‘transported’. This is helpful in terms of therapy as if a patients feels they are actually present in and experiencing an environment, they will get a similar cognitive or emotional response as if it were a real life experience, however there is still no real consensus on its formal definition or measurement [38].

Implications of dental anxiety in children and adolescents include an increased prevalence of decayed and extracted teeth, more episodes of toothache and symptomatic attendance, and negative impacts on oral health-related quality of life [39].

Conventional behavioural management techniques to decrease dental anxiety include Tell-Show-Do, desensitisation, voice control/hypnosis, applied behaviour analysis, positive reinforcement, distraction, and parental presence or absence [40]. Only a small number of these focus on home-based preparation, usually pre-teaching by parents, reading social stories, reading books about going to the dentist featuring familiar characters such as SpongeBob SquarePants (Behold, No Cavities! A Visit to the Dentist) or Dora the Explorer (Show Me Your Smile! A Visit to the Dentist), or watching similar cartoons such as ‘The Dentist’ by Peppa Pig.

Technological adjuncts usually work on the basis of distraction, and include headphones to listen to music and prevent sensory overload, playing with a tablet or watching a cartoon/film. Pharmacological adjuncts can also be considered including the use of nitrous oxide, conscious sedation or general anaesthetic. Use of these pharmacological techniques carry increased costs and associated health risks, as well as leading to reliance on general anaesthetic for treatment without addressing underlying anxiety. They are also complicated by limited access to specialist services, lengthy hospital waiting lists and cost implications associated with general anaesthetic. From every aspect, it is favourable and indeed preferable for parents to work with a child to address the underlying cause of their anxiety (for example by repeatedly using virtual reality at home, as an exposure-based approach to allow desensitisation) and facilitate conventional treatment in a general practice setting. Of course, virtual reality is not without costs, however it is hoped that by addressing the underlying anxiety the initial cost of the therapy would be significantly less than the long-term management of a dentally phobic child, perhaps requiring multiple general anaesthetics.

Many children but especially those with ASD experience dental anxiety. This can be related to fear of the unknown, difficulty communicating or reaction to sensory stimuli. This can translate to noncompliant or uncooperative behaviour. The prevalence of ASD is increasing and may be as much as 2.6% [15]. Individuals with ASD can struggle with communication, and are often quite literal, struggling with present play. They may have difficulty with change in routine and sensory awareness may be heightened. Although the traits of individuals with ASD cannot be generalised, they are often honest, observant, determined and likely to know and remember specific information [31]. The Autism Treatment Network advocates combatting any anxiety by creating a plan ahead of the visit and preparing the child, including giving them a chance to visit the dental surgery in advance (and at a quiet time), meet the team and learn the steps involved in a typical dental visit. Logically, a virtual reality environment can facilitate this preparation as well as allowing parents to repeat the preparation at their convenience. Only one of the studies identified by this review examined a cohort of patients with ASD, and simply used a phone-based application as an exposure-based approach, however no virtual reality was utilised [36]. Further research is required to examine the use of virtual reality in dentistry, however in particular in this cohort of patients who may derive the most benefit.

Behavioural theory dictates that the more a person is exposed in a safe, gentle and gradual way to a feared stimulus, the more a tolerance is built to the anxiety, which will then decrease the intensity of the fear response over time. For children with autism spectrum disorder or learning difficulties, this should be practical, visual and as close to reality as possible, as children with neurodevelopmental disorders can struggle with concepts that are abstract. It can therefore be deduced that a video or virtual reality environment demonstrating what they can expect from an experience will make it real, accessible, and reassuring, as well as giving a concrete plan and structure around appointments, all useful factors in supporting procedural anxiety.

It remains to be seen whether the use of virtual reality during dental treatment is more effective than simple audiovisual distraction, which may come without the added difficulty of a moving patient. No studies discussed the acceptability of the interventions, which may be an interesting parameter to include in further studies. In addition, the ethical implications of undertaking this form of research must be considered fully. Informed consent was reported in the studies in this systematic review but details on the exact procedures were scarce. Further high-quality research is required both around the perioperative use of virtual reality during dental treatment compared with active distraction, audiovisual distraction and filmed modelling; as well as the preoperative use of virtual reality to acclimatise or prepare patients for upcoming dental experiences.

## Conclusion

Virtual reality is a promising tool which to date has been under-utilised in dentistry. Perioperative use of virtual reality in dentistry is less ideal than in other medical procedures due to the field of interest and need for the head to remain immobile, but nonetheless further high quality trials are indicated to assess if it holds any benefit over distraction with a tablet or audiovisual glasses. High quality trials are indicated to assess the use of preoperative virtual reality and smartphone applications to prepare patients for dental examination and procedures under local or general anaesthetic.

## Clinical importance

- Medical studies have shown VR to be beneficial both as a form of distraction and acclimatisation, it follows that the same may be true in dentistry
- If children *and adolescents* can be suitably acclimatised to accept examination and treatment in an outpatient setting, a number of general anaesthetics could be avoided
- The field of VR is continuing to grow, and has innumerable applications in patient preparation, distraction, information and indeed in dental education

## Data Availability

The datasets used and/or analysed during the current study are available from the corresponding author on reasonable request. The dataset is reproducible by following the search strategy detailed in the body of the article.

## Appendix 1: Search strategy

(TITLE-ABS-KEY (medic* OR dentist* OR dental)) AND ((TITLE-ABS-KEY ((prepar* OR anxi* OR autis* OR stress*)) AND TITLE-ABS-KEY ((treatment* OR procedure* OR operation* OR "first appointment*" OR "first clinic*" OR "first attendance*" OR hospital* OR an*esthe*)))) AND ((TITLE-ABS-KEY (({app} OR app's OR apps) AND (mobile* OR smartphone* OR iphone* OR ipad* OR tablet*)) OR TITLE-ABS-KEY ({little journey}) OR TITLE-ABS-KEY ({virtual reality MRI}) OR TITLE-ABS-KEY ({take ten}) OR TITLE-ABS-KEY ({sidekicks!}) OR TITLE-ABS-KEY ({brighthearts}))) AND (LIMIT-TO (LANGUAGE, "English")) AND (LIMIT-TO (DOCTYPE, "ar") OR LIMIT-TO (DOCTYPE, "re")).

## Abbreviations

VR: virtual reality
ASD: autism spectrum disorder
GA: general anaesthetic
AV: audiovisual
MCDAS: Modified Child Dental Anxiety Scale
FLACC: Face, Leg, Activity, Cry, Consolability Scale
PECS: picture exchange communications system
